# Impact of CytoSorb Hemoadsorption Therapy on Cost-Effectiveness and Length of Stay in Critical Care Patients: A Preliminary Study from a Swiss High-Volume Center

**DOI:** 10.3390/healthcare14081103

**Published:** 2026-04-20

**Authors:** Tobias Hübner, Oliver Schöffski

**Affiliations:** 1 Departement of Anaesthesiology and Intensive Care, Kantonsspital Münsterlingen, Spitalcampus 1, 8596 Münsterlingen, Switzerland; 2 Lehrstuhl für Gesundheitsmanagement, Universität Erlangen-Nürnberg, Lange Gasse 20, 90403 Nürnberg, Germany; oliver.schoeffski@fau.de; 3 Departement of Anaesthesiology, Vögelinsegg 5, 9042 Speicher, Switzerland

**Keywords:** hemoadsorption, CytoSorb, cost-effectiveness, health economics, length of stay, DRG, reimbursement

## Abstract

**Background:** Sepsis remains a major global health challenge, associated with high mortality, prolonged intensive care unit (ICU) stays, and disproportionate healthcare costs. CytoSorb hemoadsorption offers a potential adjunct in septic shock, but real-world cost-effectiveness data in Diagnosis-Related Group (DRG)-based systems are limited. This study aimed to evaluate the clinical and economic impact of CytoSorb therapy in ICU patients with septic shock at a high-volume Swiss tertiary care center. **Methods:** A retrospective observational cohort study (2020–2023) was conducted at Kantonsspital Münsterlingen. Among 246 septic shock patients, 142 received CytoSorb therapy and 104 standard care. Patients were grouped according to treatment exposure. Baseline characteristics as well as ICU course variables, including sepsis origin, Simplified Acute Physiology Score (SAPS) II, and the Nine Equivalents of Nursing Manpower Use Score (NEMS), were compared between groups. Clinical outcomes included ICU/hospital length of stay (LOS) and duration of mechanical ventilation. Economic analysis included DRG-based revenue, direct case-related hospital costs, and net financial results. **Results:** CytoSorb-treated patients had significantly higher SAPS II scores at baseline. Despite higher initial acuity, this group showed a significantly shorter ICU LOS (median 408.5 vs. 554.5 h; *p* = 0.001), reduced hospital LOS (23.5 vs. 30.0 days; *p* = 0.008), and lower nursing workload (>20% NEMS point reduction; *p* = 0.015). Survivors treated with CytoSorb had significantly shorter ventilation durations (164.0 vs. 336.0 h; *p* = 0.014). Total hospital costs were not significantly different between groups; however, CytoSorb patients achieved a significantly better net financial result (CHF 17,125 vs. –1930; *p* = 0.025), particularly in the abdominal and pneumogenic sepsis subgroups. **Conclusions:** This study provides the first real-world evidence for the cost-effectiveness of CytoSorb hemoadsorption in septic shock, showing reduced ICU length of stay and improved financial outcomes, without increasing treatment costs or nursing workload. These findings challenge the perception of hemoadsorption as a cost driver and highlight its potential to optimize resource use in critical care. Further multicenter studies are needed to inform reimbursement strategies and integration into sepsis treatment protocols.

## 1. Introduction

Sepsis, a life-threatening organ dysfunction resulting from a dysregulated host response to infection, remains one of the most critical challenges in modern intensive care medicine [1,2]. Global estimates indicate that sepsis affects 40 to 50 million people annually, with approximately 11 million sepsis-related deaths accounting for nearly 20% of all global mortality [3]. Despite advances in antimicrobial therapy, supportive measures, and early recognition strategies, prolonged intensive care unit (ICU) stays, and subsequent complications contribute to long-term morbidity and substantial healthcare costs.

The underlying pathophysiology of sepsis is marked by a systemic inflammatory response with excessive cytokine release, commonly referred to as a ‘cytokine storm’, which may lead to multiorgan failure, prolonged ICU stays, and death if left uncontrolled [4]. In the ICU setting, patients with sepsis or septic shock frequently require complex, resource-intense care. As such, these patients are not only at high risk of poor outcomes, they also contribute disproportionately to overall hospital expenditure. In Western healthcare systems, costs associated with sepsis management now exceed those of oncology [3], while intensive care services alone represent 10–20% of total hospital costs [5]. Given the persistently high mortality and disproportionate resource consumption associated with septic shock, there is increasing interest in novel therapy approaches to improve outcomes without escalating costs. In this context, hemoadsorption has emerged as a promising adjunctive strategy to mitigate hyperinflammation. CytoSorb (CytoSorbents Europe GmbH, Berlin) is a CE-marked hemoadsorption device that can be integrated into standard renal replacement therapy, extracorporeal membrane oxygenation (ECMO) or cardiopulmonary bypass circuits, or alternatively, for stand-alone use, with a dedicated hemoperfusion pump. CytoSorb’s highly biocompatible polymer bead matrix allows for the removal of hydrophobic molecules of up to 60 kDa, including cytokines, bacterial toxins, as well as myoglobin and other compounds [6,7,8]. Its potential clinical benefits include attenuating the inflammatory response [6], stabilizing hemodynamics and reducing the need for vasopressors [9] across a wide range of indications.

Despite encouraging clinical evidence, widespread adoption of CytoSorb remains constrained by concerns around its upfront costs and reimbursement implications in Diagnosis-Related Groups (DRG)-based health systems. However, as previously suggested by Datzmann et al., in patients with high disease severity and limited prognosis, who typically fall into the high-cost segment of intensive care, the economic aspect should in principle not necessarily be viewed as the decisive factor in individual treatment decisions [10]. In Switzerland, as in many DRG-based systems, expensive technologies face intense scrutiny regarding their impact on case-based reimbursement, hospital balance sheets, and resource utilization. Given the rigid cost structures and limited bed capacities, even marginal reductions in ICU or hospital length of stay (LOS), without compromising outcomes can yield substantial economic and operational benefits and are therefore of particular interest not only to clinicians and hospital administrators, but also to healthcare payers and insurers, who seek efficient resource allocation in their shared pursuit of value-based care.

International health economic evaluations from the United States, United Kingdom, and Germany have already demonstrated favorable cost-effectiveness profiles for CytoSorb hemoadsorption, particularly in surgical patients on antithrombotic agents or for those with severe infective endocarditis [11,12,13]. These studies suggest that the upfront cost of the adsorber is offset by reductions in transfusions, complications, ICU LOS, and readmissions. However, such data are largely absent from the Swiss healthcare context, and particularly from high-volume tertiary care centers operating under the SwissDRG model, with no ICU-specific sepsis cost-effectiveness analyses published to date.

### Objective

The aim of this study was to evaluate the cost-effectiveness and impact on resource utilization of CytoSorb hemoadsorption therapy in septic shock patients treated in the intensive care unit of a high-volume Swiss tertiary care center. We used retrospective clinical and economic data and compared outcomes between patients treated with hemoadsorption and those receiving standard therapy.

Primary endpoints included DRG-based reimbursement, direct case-related hospital costs and ICU/hospital length of stay. Mortality data were also collected; however, survival analysis was not the primary focus of this study. Instead, the analysis sought to assess whether CytoSorb contributed to more efficient care delivery and optimized ICU capacity utilization in a real-world SwissDRG context.

## 2. Methods

### 2.1. Study Design and Setting

This retrospective, observational cohort study was conducted in the interdisciplinary ICU of Kantonsspital Münsterlingen, a tertiary care hospital and part of the Spital Thurgau AG group in northeastern Switzerland. The institution provides comprehensive medical services (excluding transplant medicine and neurosurgery) and manages approximately 95,000 patient days annually. The ICU is equipped with 12 beds (4 additional in reserve) and permanent mechanical ventilation capacity for up to 12 patients. The patient population includes medical, surgical, orthopedic and select gynecological and urological cases. CytoSorb hemoadsorption was formally integrated into institutional clinical practice in 2016, supported by a standardized operative procedure (SOP) outlining indication criteria and treatment protocols. As all patients included in the current analysis were treated between 2020 and 2023, the study exclusively reflects a period in which the SOP had been fully implemented and consistently applied for over four years.

### 2.2. Patient Population and Data Collection

**Figure 1:**
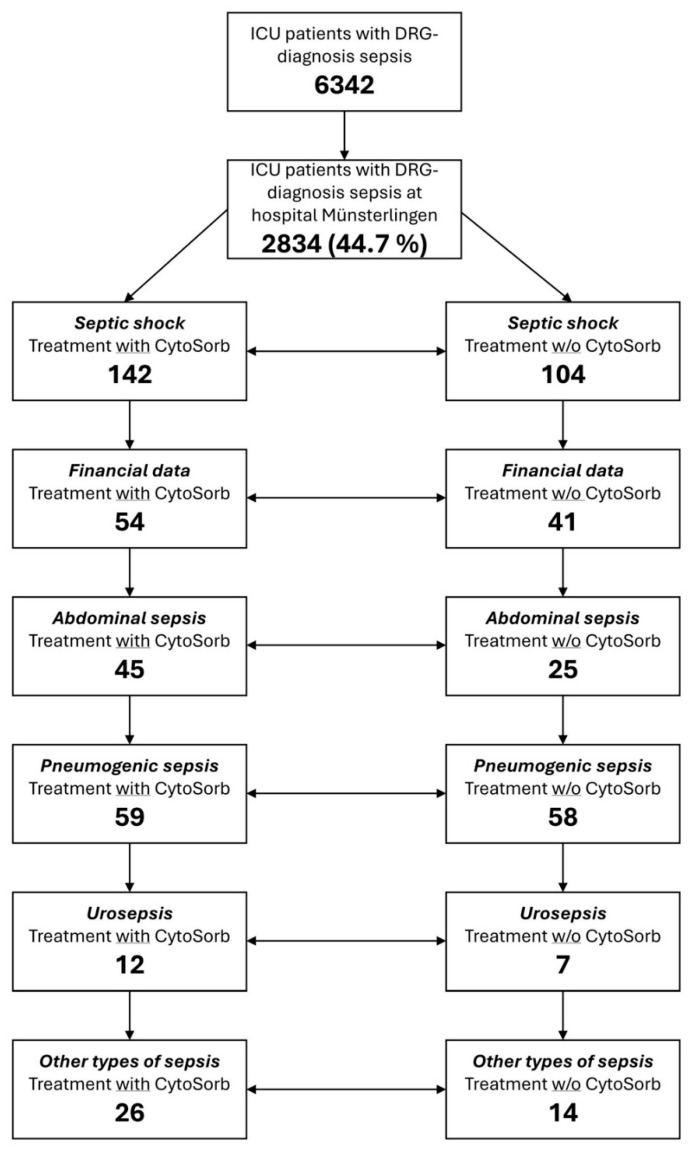
Flowchart of Patient Selection.

All adult patients (aged ≥18 years) admitted to the ICU between January 2020 and December 2023 with a primary diagnosis of sepsis or septic shock were screened for inclusion. Screening was performed retrospectively using SwissDRG-based administrative and clinical coding data. The initial screening population comprised all hospitalized patients within the Spital Thurgau hospital network coded with sepsis or septic shock as a primary diagnosis during the study period. This population was subsequently restricted to patients treated at Kantonsspital Münsterlingen and further narrowed to those admitted to the ICU with a confirmed diagnosis of septic shock, as illustrated in the patient selection flow diagram (Figure 1).

Patients treated with CytoSorb hemoadsorption during their ICU stay were identified through internal coding and clinical documentation. The indication for CytoSorb therapy followed institutional guidelines, including presence of therapy-refractory septic shock, elevated cytokine burden as defined by an Interleukin-6 (IL-6) level > 2000 ng/L and a calculated dynamic score (≥6) to support prescription of adjuvant CytoSorb hemoadsorption as described elsewhere [14]. In brief, the score was assessed within six hours of ICU admission. A score ≥ 6 prompted immediate initiation of CytoSorb therapy following standard sepsis management, independent of concurrent renal failure. Scores < 6 led to re-evaluation and rescoring after an additional six hours. While inflammatory markers (e.g., IL-6, procalcitonin) were measured, clinical presentation was the primary determinant for therapy initiation.

Treatment duration was guided by an internal calculation tool based on body weight and blood flow, which aimed for an Amount of Blood Purified (ABP) ≥ 13 L/kg. Adsorbers were routinely replaced every 12–24 h, or earlier if signs of decreased adsorption efficiency appeared, such as increasing catecholamine requirements or lactate levels. Discontinuation of CytoSorb therapy was guided by clinical stabilization and typically occurred after three adsorber cycles, unless persistent hyperinflammation or hemodynamic instability warranted continuation, following our institutional protocols which are consistent with recently published international best-practice recommendations [15].

In all cases, CytoSorb was applied via integration into continuous renal replacement therapy (CRRT) circuits. Stand-alone hemoperfusion systems, ECMO, or cardiopulmonary bypass circuits were not used for CytoSorb application during the study period.

All ICU patients with septic shock who received CytoSorb hemoadsorption during the study period were included in the intervention group. Inclusion was based on actual treatment exposure rather than post hoc confirmation of formal indication criteria. While institutional guidelines for CytoSorb use were routinely applied in clinical practice, no patients were excluded retrospectively based on adherence to specific threshold parameters.

A control group was identified from the same study period, comprising patients with septic shock who were not treated with CytoSorb and received standard care according to institutional protocols and clinical judgment, typically in cases where local criteria for hemoadsorption were not met. Baseline characteristics and ICU course variables, including sepsis origin (abdominal, pulmonary, urogenital, or other), Simplified Acute Physiology Score II (SAPS II) [16], and the Nine Equivalents of Nursing Manpower Use Score (NEMS) [17], were subsequently reported and compared between groups. Variables such as age, gender, and comorbidities were not the primary focus of this analysis, which was designed to evaluate resource utilization and cost-related endpoints within the SwissDRG framework. Furthermore, all patients were admitted with sepsis or septic shock as the primary diagnosis, ensuring a high degree of clinical comparability across cohorts. After filtering for ICU admission, diagnosis of septic shock, and availability of complete clinical and financial records, a total of 246 patients were included, with subgroup analysis performed for the 95 patients for whom cost data were available (54 with CytoSorb, 41 without). Patients treated at other hospitals, not admitted to the ICU, or without a diagnosis of septic shock were excluded during the initial cohort screening.

### 2.3. Outcomes and Variables

Patients were analyzed across three domains, i.e., clinical, economic, and operational. Clinical outcomes included ICU LOS, total hospital LOS and duration of invasive mechanical ventilation. Economic outcomes (SwissDRG-based) comprised case-level DRG revenue, case-level direct cost (excluding non-clinical overheads and non-sepsis-related interventions) and net case result (=revenue—cost). Assessed operational metrics were the SAPS II as prognostic indicator of mortality risk as well as NEMS representing a standardized nursing workload score, also used in DRG reimbursement algorithms. Of note, cost analyses were limited to the years 2022 and 2023 due to a software transition in the hospital accounting system that restricted retrospective data extraction.

### 2.4. Cost Definitions and Exclusions

Only sepsis-related, DRG-relevant direct clinical costs were included in the primary analysis (“Fallkosten 1.0”), following internal coding standards. These costs represent direct case-related clinical expenditures and do not reflect total hospital costs. Excluded cost categories were transport services, diagnostic imaging and therapies unrelated to the primary septic episode, infrastructure and energy overhead, and investments and contingency provisions. Thus, the net case result reflects a gross financial outcome based on clinical performance and DRG reimbursement parameters. All economic variables are reported as medians; therefore, net results cannot be derived by subtracting median costs from median revenues.

### 2.5. Statistical Analysis

Descriptive statistics were reported for all variables. Continuous variables including age, ventilation duration, LOS, SAPS II, NEMS, revenue, cost, and net result were summarized using mean ± standard deviation (SD), median with interquartile range (IQR), and minimum/maximum values. Age, assumed to be normally distributed, was compared between groups using a two-sample t-test. For all other continuous variables, the Wilcoxon rank-sum test was applied due to skewed distributions and the presence of outliers. Categorical outcomes, such as ICU and hospital mortality, were compared using the Chi-square test. Statistical significance was defined as *p* < 0.05 (two-sided). Data visualization included boxplots of continuous variables by treatment group and sepsis etiology, with medians and IQRs displayed. *p*-values from Wilcoxon tests and Chi-square tests were reported directly in figures where appropriate.

## 3. Results

**Figure 2:**
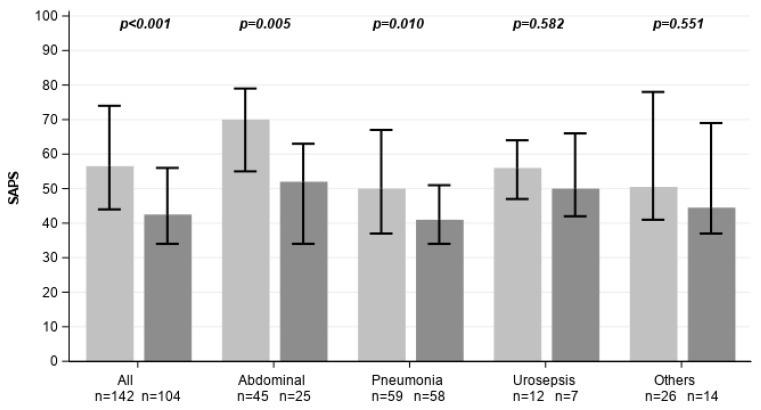
SAPS II scores across all subgroups. Light grey represents CytoSorb-treated individuals, dark grey control patients. Data are shown as medians with error bars indicating the interquartile range (Q1–Q3).

Over the four-year observation period (2020–2023), a total of 6342 hospitalized patients received the sepsis code as one of the relevant main diagnoses across both acute care hospitals within the Spital Thurgau network. Of these, 2834 patients (44.7%) were treated at Kantonsspital Münsterlingen. From this population, a total of 246 ICU patients with sepsis were included in the study, of whom 142 received CytoSorb hemoadsorption therapy and 104 were managed with standard of care (SoC) alone (Figure 1). The cohort was categorized into four etiological subgroups: pneumogenic sepsis (n = 117), abdominal sepsis (n = 70), urosepsis (n = 19), and sepsis of other origins (n = 40). Group distributions were balanced within each subgroup. Financial data (case costs, DRG revenue, net result) were available for 95 patients (54 CytoSorb, 41 SoC), representing 38.6% of the total cohort. There were no significant differences in mean age between the two treatment groups. However, patients treated with CytoSorb had significantly higher SAPS II scores across all subgroups, reflecting a more critical condition on ICU admission and a greater predicted risk of mortality (Figure 2, Table 1).

### 3.1. Nursing Effort (NEMS)

Despite higher initial acuity, patients in the CytoSorb group demonstrated a significantly lower cumulative nursing workload on the ICU, as reflected by a >20% reduction in total NEMS points (1624.5 vs. 2054.5; *p* = 0.015). This trend persisted across most subgroups. No significant difference in NEMSs was observed in pneumogenic sepsis (Table 1).

### 3.2. Length of Stay and Mechanical Ventilation

**Figure 3:**
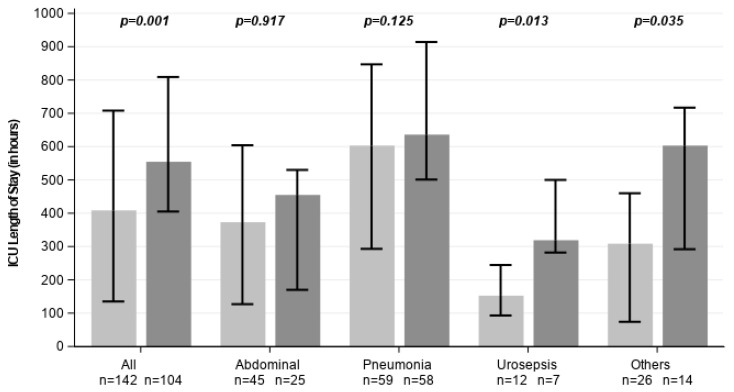
ICU length of stay across all subgroups. Light grey represents CytoSorb-treated individuals, dark grey control patients. Data are shown as medians with error bars indicating the interquartile range (Q1–Q3).

CytoSorb therapy was associated with a marked reduction in ICU and hospital LOS (Figure 3). Median ICU LOS was significantly shorter in the CytoSorb group compared to SoC (408.5 vs. 554.5 h; *p* = 0.001). Among survivors, ICU LOS in CytoSorb-treated patients was considerably shorter compared to controls, although not statistically significant (480.0 vs. 634.0 h; *p* = 0.096, Table 1).

**Figure 4:**
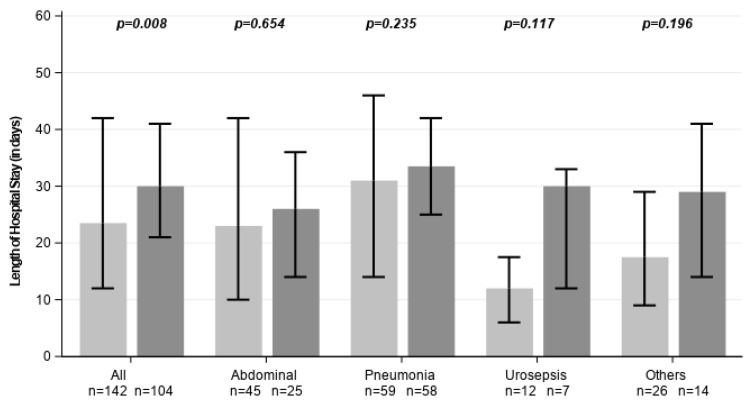
Hospital length of stay across all subgroups. Light grey represents CytoSorb-treated individuals, dark grey control patients. Data are shown as medians with error bars indicating the interquartile range (Q1–Q3).

Hospital LOS was also shorter (23.5 vs. 30.0 days; *p* = 0.008), although subgroup-specific differences did not reach statistical significance (Figure 4).

**Figure 5:**
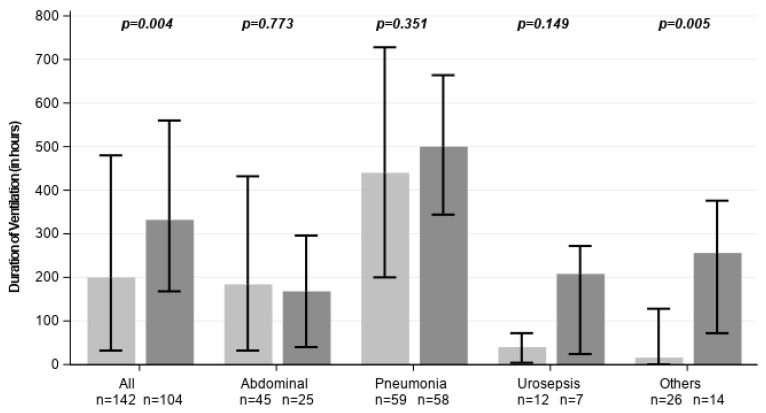
Duration of mechanical ventilation across all subgroups. Light grey represents CytoSorb-treated individuals, dark grey control patients. Data are shown as medians with error bars indicating the interquartile range (Q1–Q3).

Total ventilation duration was significantly shorter in the CytoSorb group across the entire study cohort and among survivors (Figure 5). In patients who ultimately survived, median duration of mechanical ventilation was significantly shorter in the CytoSorb group compared to control individuals (164.0 vs. 336.0 h; *p* = 0.014, Table 1). In patients who died during hospitalization, no significant difference in ventilation duration was observed between groups. Notably, patients with abdominal sepsis in the CytoSorb group required non-significantly longer ventilation.

### 3.3. Economic Outcomes

Contrary to expectations, total hospital case costs were not significantly higher in the CytoSorb group, even though these patients were more critically ill and incurred the additional costs of the CytoSorb devices (Table 2). Among survivors, a trend toward higher costs was observed, not reaching statistical significance. Importantly, no cost disadvantage was identified for CytoSorb in any subgroup or outcome scenario.

While overall DRG revenue per case did not differ significantly between groups, a favorable trend toward higher reimbursement was noted in CytoSorb-treated patients with urosepsis (Table 2). Among non-survivors, SoC patients generated the highest DRG income.

**Figure 6:**
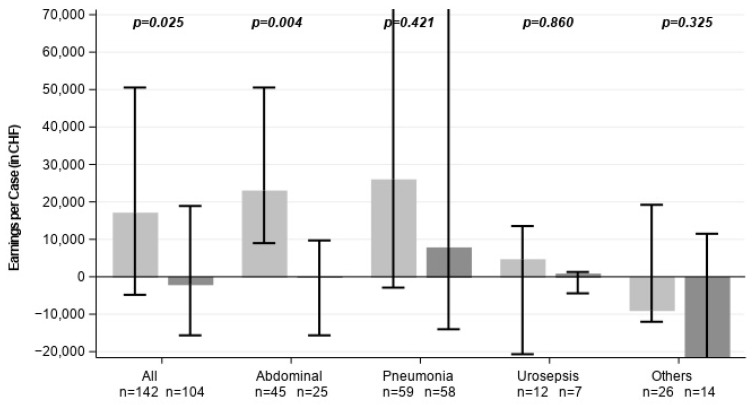
Earnings per case (net case profitability) with and without CytoSorb hemoadsorption (in CHF). Light grey represents CytoSorb-treated individuals, dark grey control patients. Data are shown as medians with error bars indicating the interquartile range (Q1–Q3).

Net case profitability (revenue minus costs) showed a clear advantage for CytoSorb therapy (Table 2). The overall cohort demonstrated significantly higher earnings per case for CytoSorb patients compared to SoC (17,125 Swiss Franc [CHF] vs. –1930 CHF, *p* = 0.025, Figure 6). This difference showed a non-significant trend in the subgroup of survivors (*p* = 0.09) and remained significant in abdominal sepsis patients. In other subgroups, there was a trend towards increased net profit in the CytoSorb group, although significance was not reached likely due to the small sample sizes and variance.

### 3.4. Mortality

As expected from their higher SAPS II scores, CytoSorb patients had a higher overall mortality, both in the ICU and with regard to the entire hospitalization period (Table 1). While not a primary endpoint of this study, mortality was analyzed to contextualize economic and LOS outcomes. The increased mortality is interpreted as a reflection of patient severity rather than therapy effect and is further addressed in the discussion.

**Table 2:** Economic outcomes (SwissDRG-based).

	Overall Cohort			Abdominal			Pneumogenic			Urosepsis			Miscellaneous			Non-Survivors			Survivors		
	CytoSorb (n = 54)	SoC (n = 41)	*p*-Value	CytoSorb (n = 19)	SoC (n = 14)	*p*-Value	CytoSorb (n = 19)	SoC (n = 18)	*p*-Value	CytoSorb (n = 4)	SoC (n = 3)	*p*-Value	CytoSorb (n = 12)	SoC (n = 6)	*p*-Value	CytoSorb	SoC	*p*-Value	CytoSorb	SoC	*p*-Value
**Cost per case [CHF]**	−9.786 [−11,313.0, −7500.0]	−9.119 [−10,721.0, −7323.0]	0.350	−9.629 [−11,096.0, −7473.0]	−8.681 [−10,078.0, −7867.0]	0.813	−8.408 [−10,958.0, −6846.0]	−8.952 [−10,904.0, −5555.0]	0.820	−10.023 [−13,335.5, −8415.5]	−11.151 [−12,769.0, −9449.0]	0.860	−11.940 [−14,755.0, −8807.5]	−8.937 [−12,819.0, −7163.0]	0.281	−9.495 [−11,434.0, −7500.0]	−8.737 [−10,551.0, −7895.0]	1000	−10.044 [−11,171.0, −8109.0]	−9.308 [−10,904.0, −6121.0]	0.285
**DRG revenue per case [CHF]**	124,423 [61,111.0, 223,220.0]	125,762 [99,802.0, 173,923.0]	0.819	159,806 [86,168.0, 267,817.0]	164,655 [77,721.0, 195,345.0]	0.548	145,826 [78,519.0, 337,665.0]	158,965 [103,235.0, 173,923.0]	0.891	56,799 [48,986.5, 63,591.0]	53,885 [40,206.0, 118,566.0]	0.860	89,220 [35,731.5, 203,511.0]	110,936 [98,189.0, 154,972.0]	0.888	95,242 [57,603.0, 185,624.0]	172,639 [46,753.0, 231,957.0]	0.536	145,826 [77,541.0, 228,269.0]	125,363 [100,847.0, 166,191.0]	0.426
**Earnings per case [CHF]**	17,125 [−4818.0, 50,542.0]	−1.930 [−15,644.0, 18,908.0]	0.025	23,037 [−60,033, 150,432]	77 [−71,340, 44,908]	0.004	26,026 [−2904.0, 86,902.0]	7843 [−14,017.0, 84,519.0]	0.421	4699 [−20,682.5, 13,539.0]	849 [−4422.0, 1268.0]	0.860	−8.848 [−12,023.0, 19,219.5]	−22.066 [−50,255.0, 11,493.0]	0.325	16,259 [−1223.0, 48,726.0]	6863 [−9764.0, 24,283.0]	0.229	17,991 [−6508.0, 55,211.0]	−2.364 [−21,371.0, 18,908.0]	0.091

Depicted are median values and IQR [Q1, Q3] or percentage (%) as appropriate.

## 4. Discussion

This retrospective analysis evaluated the economic and clinical impact of CytoSorb hemoadsorption therapy in septic shock patients treated in a real-world Swiss ICU setting. From a total of 246 ICU patients with septic shock, the use of CytoSorb hemoadsorption therapy was associated with trends toward a reduction in ICU and hospital length of stay, significantly lower nursing workload (as measured by NEMS), and a neutral to improved cost profile, despite higher baseline illness severity in the intervention group. Importantly, use of hemoadsorption did not lead to higher treatment costs and the net financial result per case was better in the CytoSorb compared to the standard-of-care group, despite the fact that patients treated with CytoSorb had higher baseline severity of illness, as indicated by significantly elevated SAPS II scores. However, as a retrospective observational analysis, these findings must be interpreted with caution and do not establish definitive causal relationships. To the best of our knowledge, this is the first study to evaluate both clinical and economic endpoints of CytoSorb therapy in a real-world critical care environment, an increasingly important perspective amid rising cost pressures in intensive care units.

These findings align with prior economic evaluations, such as the UK-based cost-utility analysis by Javanbakht et al. [12], which demonstrated reduced costs and improved quality-adjusted life years (QALYs) in patients receiving CytoSorb during cardiac surgery aimed at ticagrelor reversal.

Assessing cost-effectiveness in intensive care medicine remains inherently challenging [18]. The variability in patient acuity, unpredictable disease trajectories, and the presence of extreme outliers as cost drivers make traditional economic tools such as DRG data, demographic metrics, and severity scores only partially applicable. While extreme outliers as cost drivers can indeed be identified and excluded to smooth the data set, doing so inevitably draws attention to precisely these high-cost cases that reinforce the perception of intensive care medicine as inefficient and overly expensive.

In this context, health economic studies such as that by Cohen et al. have underscored the importance of evaluating CytoSorb’s potential to mitigate bleeding-related complications and hospital resource utilization in cardiac surgery, supporting the potential broader rationale for hemoadsorption’s economic viability across indications [11].

In our cohort, DRG revenues were similar between groups, which was expected given that sepsis treatment is protocol-driven and largely standardized. However, this finding is of outmost importance, as CytoSorb does not inflate costs by virtue of its use alone, but rather contributes to improved net financial outcomes, compared to standard therapy. This applies to the overall cohort as well as to both deceased and surviving patients. These findings support the principles of value-based healthcare by demonstrating that a targeted therapy can improve outcomes and resource utilization without increasing costs [19].

Similar trends have been reported by Rao et al., who conducted a German budget impact analysis and observed improved financial outcomes in patients with infective endocarditis treated with CytoSorb, further supporting its potential cost-effectiveness in high-risk, resource-intensive clinical settings [13].

When analyzing the data in the context of nursing workload (NEMS) and disease severity (SAPS II), patients treated with CytoSorb were more critically ill compared to those receiving standard care. Despite this, their care requirements as measured by nursing effort were not significantly elevated. This finding suggests that hemoadsorption may facilitate more rapid clinical stabilization, effectively offsetting the procedural complexity introduced by extracorporeal circuit integration. Notably, the observed reduction in cumulative NEMS points exceeding 20% is not merely a statistical artifact but translates into meaningful resource implications. Based on prior work correlating NEMSs with direct nursing effort, each NEMS point approximates 56 min of care within a 24-h ICU period [20]. Applying this benchmark, the difference observed in our cohort equates to several hundred hours of cumulative nursing time saved in the hemoadsorption group, highlighting a potential operational benefit and staff resource relief that extends beyond clinical stabilization alone. Furthermore, the absence of a measurable increase in nursing demand attributable to potential confounding factors, such as comorbidities, supports the concept that CytoSorb delivers clinical benefits without imposing additional strain on ICU resources. Notably, while hemoadsorption is currently not reimbursed as a dedicated add-on within the SwissDRG framework, dialysis therapies are eligible for supplementary compensation. When applied in combination, these therapy-related surcharges may enhance the overall economic viability of hemoadsorption in intensive care settings.

One of the clearest economic advantages emerged in patients with abdominal and pneumogenic sepsis, most probably due to a shorter ICU and total hospital stay in these patients, resulting in increased revenues and decreased costs. This timing is critical, as DRG reimbursement is optimized when discharges occur near the mean length of stay. Patients discharged before the upper threshold avoid revenue penalties associated with prolonged stays. In abdominal sepsis, the paradoxical finding of longer mechanical ventilation in CytoSorb patients is likely explained by a higher rate of reoperations, necessitating continued sedation for procedural planning rather than ongoing organ failure. These patients still had shorter ICU stays overall, contributing to a better financial margin. Importantly, the observed difference in ventilation time was marginal and may therefore be interpreted as clinically acceptable. As such, shorter ICU and hospital lengths of stay allow hospitals to avoid reaching upper DRG thresholds and enable faster bed turnover. Consequently, fixed costs can be distributed over a larger patient base, while freeing up capacity for new admissions.

Although mortality was not a primary endpoint of this study, it warrants brief consideration. Importantly, given the retrospective cohort design without formal matching or adjustment, differences in mortality between groups are expected and reflect real-world treatment allocation rather than balanced group comparability. As expected, the CytoSorb group had higher ICU and hospital mortality rates (50.7% vs. 41.3% in the SoC group, not statistically significant), largely due to the selection of patients with increased disease severity and limited alternative treatment options. In clinical practice, CytoSorb is often employed relatively late in the disease course in patients who fail to respond to standard interventions. This is particularly relevant given the lack of strong guideline recommendations for hemoadsorption in septic shock, categorizing its use as an “individualized therapeutic attempt” rather than standard of care. Still, its ability to promote early hemodynamic stabilization aligns with core treatment goals outlined in current sepsis guidelines [21,22]. Thus, mortality outcomes are confounded by indication bias rather than therapy efficacy. The only subgroup with statistically significant increased hospital mortality in the CytoSorb group was pneumogenic sepsis, a finding likely influenced by the disproportionate burden of Coronavirus disease 2019 (COVID-19) cases during the study period. COVID-19 related septic shock is known to carry a higher mortality rate than bacterial pneumonia or other respiratory pathogens. While the pandemic context may limit generalizability, it also underscores the relevance of CytoSorb as a potential intervention in high-risk, hyperinflammatory states where standard therapies often fail. At this point it should be noted, however, that retrospective differentiation between viral (COVID-19) and bacterial pneumonias (primary or possible superinfection) was not systematically possible in our dataset, and mortality analyses should be interpreted in the light of this diagnostic overlap. Overall, mortality differences between groups likely reflect residual confounding and indication bias inherent to retrospective observational designs and should not be interpreted as evidence of treatment harm or benefit. As such, these results are rather hypothesis-generating than definitive and support the design of prospective multicenter trials focused on the health economic impact of hemoadsorption therapy in septic and other critically ill populations. Moreover, although this study focused on in-hospital outcomes, the observed reductions in ICU burden and clinical workload may also translate into long-term savings through reduced morbidity and post-ICU rehabilitation needs, areas warranting further investigation. It should be noted that this cohort was treated during a period preceding the publication of controversial results from some randomized trials, and institutional practice was guided by the evidence and clinical experience available at that time. Future research may also consider non-inferiority approaches to substantiate clinical equivalence and assess long-term outcomes, with an additional focus on demonstrating measurable gains in resource utilization and operational efficiency.

### 4.1. Limitations

This analysis is subject to several limitations inherent to its retrospective, single-center design and the data structure within the Swiss healthcare system. First, all data were collected from ICU patients at Kantonsspital Münsterlingen and are therefore impacted by local processes, staffing models, and institutional workflows. As such, the findings may not be directly transferable to ICUs in hospitals of different sizes, service levels, or infrastructure—particularly university hospitals or tertiary referral centers. Second, cost data in this study are assigned on a per-case basis and linked to intensive care via DRG classification and Swiss Classification of Surgical Interventions (CHOP) coding. While this allows for standardized economic analysis, it introduces variability: higher case costs do not necessarily reflect increased material or personnel input. For instance, patients who remain hospitalized while awaiting transfer to a rehabilitation facility continue to generate DRG-based costs without proportionate resource utilization, particularly if their stay exceeds the upper DRG threshold. This reimbursement dynamic differs substantially from systems like that in Germany, for, e.g., where patients awaiting rehab are typically transferred to intermediate care or discharged home.

Given the retrospective design, no formal matching or multivariable adjustment was performed. Therefore, residual confounding and selection bias cannot be excluded.

Personnel costs also present a limitation. Although the use of CytoSorb necessitates an extracorporeal circuit, typically managed by more highly qualified (and higher-cost) ICU nursing staff, these personnel costs are pooled and distributed across all ICU cases rather than being assigned by skill level or treatment modality. As a result, specific differences in nursing qualifications and deployment intensity could not be adjusted for in the economic analysis.

Furthermore, the heterogenetity of the patient population limits the precision of cost attribution. Many ICU patients included in this study presented with multiple comorbidities, which may have influenced both clinical decision-making and incurred costs unrelated to the primary diagnosis of sepsis or to CytoSorb therapy itself. These costs are nonetheless allocated to the overall intensive care episode, potentially diluting the specific cost signal related to hemoadsorption.

The accuracy of diagnostic grouping also poses a methodological challenge. Within the hospital’s financial and coding software, the principal diagnosis assigned for billing purposes may not reflect the actual reason for ICU admission. Consequently, the classification of patients into specific sepsis subtypes (e.g., pneumogenic vs. abdominal) required manual chart review and verification. While this improved categorization accuracy, it introduces a degree of subjectivity and potential classification bias.

Because death acts as a competing event for discharge, we used survivor-stratified analyses to mitigate mortality confounding; competing-risk/time-to-discharge methods (e.g., Kaplan–Meier-based approaches) and ‘days alive’ endpoints were not applied and should be considered in future prospective studies.

Lastly, mortality significantly influences both length of stay and economic outcomes. This was particularly relevant in the subgroup of patients with pneumogenic sepsis, many of whom were treated during the COVID-19 pandemic. These patients exhibited markedly higher mortality rates and shorter lengths of stay compared to non-COVID cohorts. According to our internal benchmarking, the observed ICU mortality rates remain within international ranges. To account for this variable, all data were stratified and analyzed separately for survivors and non-survivors. However, mortality may act as a competing event influencing both length of stay and cost, as early death can reduce overall resource utilization. Competing-risk or time-to-event approaches were not applied in this retrospective analysis and should be considered in future studies.

### 4.2. Conclusions

In addition to previous economic evaluations primarily centered on cardiac surgery indications, this study offers preliminary real-world data on the potential cost-effectiveness of CytoSorb hemoadsorption in ICU patients with septic shock, one of the most resource-intense populations in modern hospital care. Therapy was associated with financial advantage by reducing ICU length of stay and enabling more efficient bed turnover without increasing treatment costs or nursing workload. As such, CytoSorb appears to improve operational and economic outcomes, directly countering the perception of hemoadsorption as a cost driver. These early findings provide an important foundation for both future research and for policy-level decisions on the integration of hemoadsorption therapies into sepsis management protocols and reimbursement models. Future multicenter studies across various healthcare systems are needed to further assess the health economic impact of hemoadsorption and guide evidence-based reimbursement strategies.

## Figures and Tables

**Table 1:** Baseline characteristics as well as operational metrics and clinical outcomes.

	Overall Cohort			Abdominal			Pneumogenic			Urosepsis			Miscellaneous			Non-Survivors			Survivors		
	CytoSorb (n = 142)	SoC (n = 104)	*p*-Value	CytoSorb (n = 45)	SoC (n = 25)	*p*-Value	CytoSorb (n = 59)	SoC (n = 58)	*p*-Value	CytoSorb (n = 12)	SoC (n = 7)	*p*-Value	CytoSorb (n = 26)	SoC (n = 14)	*p*-Value	CytoSorb	SoC	*p*-Value	CytoSorb	SoC	*p*-Value
**Age [years]**	67.0 [58.0, 76.0]	69.0 [61.0, 76.0]	0.163	74.0 [64.0, 81.0]	78.0 [66.0, 83.0]	0.287	65.0 [58.0, 75.0]	68.0 [59.0, 73.0]	0.672	69.5 [59.0, 74.0]	69.0 [63.0, 76.0]	0.322	59.0 [44.0, 70.0]	65.5 [57.0, 75.0]	0.187	72.0 [61.0, 79.0]	73.0 [66.0, 80.0]	0.355	65.0 [56.0, 71.0]	67.0 [59.0, 73.0]	0.095
**SAPS II [points]**	56.5 [44.00, 74.00]	42.5 [34.00, 56.00]	<0.001	70.0 [55.00, 79.00]	52.0 [34.00, 63.00]	0.005	50.0 [37.00, 67.00]	41.0 [34.00, 51.00]	0.010	56.0 [47.00, 64.00]	50.0 [42.00, 66.00]	0.582	50.50 [41.00, 78.00]	44.5 [37.00, 69.00]	0.551	67.00 [50.00, 79.00]	51.0 [39.00, 63.00]	<0.001	49.5 [37.00, 67.00]	41.0 [33.00, 50.00]	0.012
**NEMS [points]**	1624.5 [572.0, 3047.0]	2054.5 [1565.0, 2918.0]	0.015	1487.0 [549.0, 2699.0]	1551.0 [593.0, 1925.0]	0.686	2503.0 [1465.0, 3713.0]	2445.5 [1970.0, 3521.0]	0.525	553.5 [331.0, 813.0]	1529.0 [793.0, 1783.0]	0.038	1070.5 [324.0, 1739.0]	1988.5 [1211.0, 2656.0]	0.026	1476.5 [363.5, 2841.5]	1984.0 [1529.0, 2885.0]	0.059	1690.5 [837.0, 3409.0]	2117.0 [1653.0, 3069.0]	0.168
**Length of ventilation [hours]**	200 [32.0, 480.0]	332 [168.0, 560.0]	0.004	184 [32.0, 432.0]	168 [40.0, 296.0]	0.773	440 [200.0, 728.0]	500 [344.0, 664.0]	0.351	40 [4.0, 72.0]	208 [24.0, 272.0]	0.149	16 [0.0, 128.0]	256 [72.0, 376.0]	0.005	216 [40.0, 532.0]	328 [152.0, 550.0]	0.110	164 [16.0, 448.0]	336 [200.0, 560.0]	0.014
**Length of ICU stay [hours]**	408.5 [135.0, 708.0]	554.5 [405.0, 809.0]	0.001	373 [127.0, 604.0]	455 [170.0, 530.0]	0.917	603 [293.0, 847.0]	636 [501.0, 914.0]	0.125	152.5 [93.0, 244.5]	319 [282.0, 500.0]	0.013	308.5 [74.0, 460.0]	603 [292.0, 717.0]	0.035	306 [73.5, 599.0]	475 [311.0, 614.0]	0.011	480 [255.0, 918.0]	634 [476.0, 867.0]	0.096
**Length of hospital stay [days]**	23.5 [12.0, 42.0]	30 [21.0, 41.0]	0.008	23 [10.0, 42.0]	26 [14.0, 36.0]	0.654	31 [14.0, 46.0]	33.5 [25.0, 42.0]	0.235	12 [6.0, 17.5]	30 [12.0, 33.0]	0.117	17.5 [9.0, 29.0]	29 [14.0, 41.0]	0.196	16 [7.0, 26.0]	25 [17.0, 33.0]	0.007	36 [17.0, 53.0]	36 [27.0, 48.0]	0.792
**ICU Mortality [%]**	45.8	23.1	<0.001	44.4	16.0	0.016	57.6	31.0	0.004	25.0	14.3	0.581	30.8.	7.1.	0.088						
**Hospital Mortality [%]**	50.7	41.3	0.146	53.3	44.0	0.454	59.3	37.9	0.021	25.0	57.1	0.161	38.5	42.9	0.787						

Depicted are median values and IQR [Q1, Q3] or percentage (%) as appropriate.

## Data Availability

The raw data supporting the conclusions of this article will be made available by the authors on request.

## List of Abbreviations

CHF	Swiss Franc
CHOP	Swiss Classification of Surgical Interventions
COVID-19	Coronavirus disease 2019
DRG	Diagnosis Related Groups
ECMO	Extracorporeal membrane oxygenation
ICU	Intensive Care Unit
IL-6	Interleukin-6
LOS	Length of stay
NEMS	Nine equivalents of nursing manpower use score
PCT	Procalcitonin
QALYs	Quality-adjusted life years
SAPS	Simplified Acute Physiology Score
SoC	Standard-of-care
